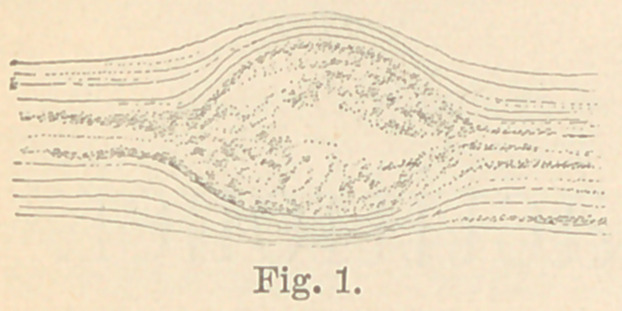# A Discussion of Some Questions in Dental Caries

**Published:** 1885-04

**Authors:** W. D. Miller

**Affiliations:** Berlin, Germany


### Independent Practitioner.

Vol. VI. April, 1885. No. 4.

### Original Communications.

### A DISCUSSION OF SOME QUESTIONS IN DENTAL CARIES.

BY W. D. MILLER, BERLIN, GERMANY.

Since the publication of the results of my investigations in the etiology of dental caries, they have been the object of an unusual amount of discussion, and not a few, who in every sentence betray their utter unfitness for the work they have undertaken, have felt themselves called upon to point out my mistakes, and to present what they are pleased to call the logical deductions from my experiments. Moreover, sentences have been picked out without any regard to their connection or date of publication, and put together in the manner which appeared best suited to the wants of the speaker; or, with striking liberality, statements are attributed to me which will be found contradicted on almost every page of my writings. The dicussions of this subject in the different dental societies, have shown a lack of knowledge on the part of many who assert their right as teachers, which is not very creditable to them or the profession of which they are members. It is not my intention to reply to anything of this nature which has appeared in the dental journals. There are a few other points, however, which I will notice briefly.

The question has been raised as to the causes of the distention of the dental tubuli, seen in caries. I state, then, that micro-organisms *can*, and often *do* not only distend separate tubules, but push whole tubules aside. (See Fig 1.) I have examined too many sections to be deceived in this; nor is there anything strange in it. We know that bacteria are unaffected by pressures far above the normal; we sometimes see, moreover, capillary blood vessels varicose and distended to many times their natural lumen, by the masses of micro-organisms.

Fig. 1.

"S.," in the *New England Journal of Dentistry*, tells us that "Dr. Miller seems to account for 'this phenomena' by the simple action of lactic acids." Now if there be anything which is specially offensive, it is to have some would-be critic thus tamper with the results of actual experiments, and I must beg "S." for the future to allow me to promulgate my own views. There is not a sentence in all that I have ever written which would justify this statement. The manner in which the further widening of the tubules, the breaking down of the basis-substance and the formation of caverns takes place, is concisely put in the *Dental Cosmos* for 1883, page 339, where I say: "The micro-organisms commonly found in the human mouth, are capable of readily transforming such softened dentine into substances suitable to their nourishment; in other words, of consuming it." Again, in the INDEPENDENT PRACTITIONER, 1883, p. 64: "There is no expansion of the tubules, no formation of caverns, no melting down of the tissue * * * * * * * * where fungi are not present." The manner in which this takes place was further set forth in a paper read before the Central Verein deutscher Zahnarzte, in Berlin, Aug. 3d, 1884. I then said: "If we throw a piece of boiled white of egg into a culture of the hay bacillus (*Bacillus subtilis*), it will gradually become smaller, and in a few days disappear; or if we add a piece of decalcified dentine to a culture of caries-fungi, it will in a like manner be dissolved. The same thing takes place in caries of the teeth. The same micro-organisms which produce an acid reaction of the fluids in a cavity of decay, possess the power, either directly or by means of a ferment which they produce, of dissolving the decalcified dentine." Either of the above quotations seems to present the case with sufficient clearness. The fungi, demonstrably and as I have *proved* by the experiment cited above, do possess the power of dissolving the decalcified dentine, or digesting it, if you please; but not a single fact can as yet be produced to show that they can soften *normal* dentine, either directly or by means of any ferment which they produce. In fact, the only experiments which have ever been made at all, point directly to the opposite conclusion. I have exposed pieces of dentine for years to the action of micro-organisms in non-fermentable solutions, without producing the slightest softening.

We would pay very little credit to the labors of a man who, not having mastered addition and subtraction, would attempt to solve a problem in calculus, yet there are not a few of our profession who, without knowledge of the simplest principles of bacteriological investigation, or without the least experience in bacteriological experimentation, do not hesitate to present a solution of the most difficult problems. Dentists get up in their various societies and remark that they have "repeated the experiments of Dr. Miller, and have not obtained the same results." Milles and Underwood, who have done more work of this nature than any American experimenter, if I may not say more than all American experimenters together, and whose communications are entitled to respect, reported that they had repeated my experiments; they were, however, consistent enough with the practice of scientific investigators to give in detail the manner in which they did it, and it was at once evident that their experiments were as unlike mine as they could well be. Consequently, I cannot be expected to consider it as a very serious matter when Quisdam chooses to get up in a dental society, and say that he has repeated my experiments without obtaining the same results.

I have proved that the softening of the dentine in caries is nothing more nor less than a simple decalcification; that the acids by which it is produced may be readily detected about decaying teeth; that these acids are formed by the action of certain micro-organisms, which, *being independent of the presence of air*, may form acids within the substance of the dentine; furthermore, that the same fungi have the power of dissolving the softened dentine. There is not a step in the process which has not been proved and made clear by repeated experiments, and which does not suggest its own reasonableness to every impartial practitioner. Finally, I have produced artificial caries which the best authorities on microscopy cannot distinguish from natural caries. Nevertheless, there are those who insist on the action of some unnatural agent or agents, for whose existence they cannot offer a shadow of proof, just as a few years ago some would have it that caries is the result of electrical action between the different tissues of the tooth, or between the tooth-bone and filling, although no one was ever able to detect the culpable electricity, and although by the very nature of things it could not exist.

The Springfield school of "logicians," a short time ago, would hear of nothing but putrefaction, while the combined results of all the experimenters and observers of the world plainly told them that no one ever did or ever could produce caries by putrefaction alone. So "S." hypothecates the existence of a particular toxic agent, and, having done that, he hypothecates to his agent the power of softening dentine, while no one has ever detected the agent, much less produced caries by its action.

I have shown that the fungi of dental caries produce, first an inverting ferment, and secondly an acid-producing ferment, and thirdly (by experiments soon to be published) a peptonizing ferment. I have furthermore shown that there is a fungus in the human mouth, which, when injected into the blood vessels of small animals, produces blood-poisoning, demonstrating that it does, no doubt, produce a toxic agent, but this certainly would not justify the inference that all or any of the fungi of caries produce toxic agents, or that such agents have any effect upon sound dentine.

With regard to the digestive fluid, the avidity with which the mention of it is seized reminds us of the Bourgeois gentilhomme: "What! when I say, 'Nicole, bring me my slippers and give me my night-cap,' is that prose? Upon my honor, then, I have been talking prose these forty years, and have not known it." Toxic agents! digestive fluid! they exclaim; we never heard of them before; they must be the cause of dental caries. It does not matter whether any of the fungi of dental caries really produ e toxic agents or not, or whether if they did, tooth-bone would be affected by them; here is something (old) which to us is new; here must be the explanation of all caries. They even go so far as to speak of *Dr. Black's* digestive fluid. Dr. Black certainly does not claim to be the first to detect the existence of such an agent. Will some one please name an organism with a digestive fluid (not acid) sufficiently strong to digest dentine or enamel not previously decalcified?

When we know that we have so many micro-organisms in the human mouth, and know that many of them are continually producing acids there, and know that this acid very readily attacks and softens dentine (or enamel), and know that they are capable of dissolving the softened dentine, then why not hold fast that we *know*, instead of rushing headlong after something that we know nothing about?

It would seem as though certain of my contemporaries had consecrated themselves to the sole object of putting everything possible in the way of a clear understanding of the subject, of distorting and confusing the simplest observations, and substituting for them anything that can be seized upon, utterly regardless of its complete incoherence.

It is not very long since the view was strenuously advocated in the *New England Journal*, that caries does not begin until putrefaction of some portion of the organic matter sets in. Now, Mayr tells us that "caries comprises only that small diseased layer, probably not over one-one-hundredth inch thick, that was sensitive." It would be difficult to say which of these views has less foundation in fact. The absurdity of making sensitiveness the test of caries must be at once apparent to every practitioner, even if we do not take into account pulpless and dead teeth. Equally non-accordant with the most simple facts of histology is the comparison of the mass of carious dentine to a scab; there is not the slightest analogy between the two, nor is there between pus and the outer layers of carious dentine. Such misleading terms should be scrupulously avoided. It is very plausible to make a theory of dental caries, and then frame a definition of caries which will conform to the theory; it does everything else, however, more than it advances the cause which it pretends to serve.

It has been, furthermore, persistently asserted in the *New England Journal*, that in that portion of the softened dentine bordering on the normal the lime salts are present in normal proportions, whereas analyses of this layer, made by Dr. Jeserich, and by myself, have shown a very considerable reduction in the proportion of lime salts. (See *Cosmos*, 1883, p. 342.) As I pointed out in the article just referred to, the analyses on which this assertion is based having been undertaken with one milligram of carious dentine, were necessarily completely unreliable, as every dentist in the United States may find out by enquiring of any competent authority in chemistry. Since my connection with the Dental Institute, I have had abundant opportunity to secure the material necessary for carrying out these analyses, and the result has completely confirmed the previous work.

From about one hundred and fifty teeth, I chose thirty of those best adapted, removed all the outer, very soft dentine, and took for the purpose of analysis only a very thin layer on the border of the normal dentine. The instrument grated on the hard dentine, and the almost or completely colorless dentine came off in little chips, great care being taken that no trace of enamel should get mixed in with the dentine. The analysis made by Dr. Jeserich, a sworn chemist, the highest authority here, gave sixty per cent. of lime salts, while a similar analysis made by myself gave sixty-one per cent. As the amount of dentine used in the determinations was in each case above 75 mg, and the analyses were made with great care, it may be safely said that the error of experiment fell within one and one-half to two per cent. at the outermost. Herr Bennefeld (*Correspondenz Blatt fur Zahnarzte,* Jan., 1885) found in an analysis of over 100 mg., obtained from about fifty teeth, sixty and four-tenths per cent. of lime salts. These results indicate the exhaustion of over one-third of the lime salts. Were it true that the softening of the dentine on the border line is produced by a destruction of the organic matter, these analyses should show an *increase* in the percentage of lime salts, a result which no one has ventured to publish, and how, in the face of this fact, any school of *philosophers* could persistently adhere to and promulgate such a doctrine, is more than I can understand. If this be logic, then let us "throw" logic "to the dogs." Moreover, the necessary experiments have not yet been made to prove that those who make such a parade of logic know anything more about it than the lookers-on.

I have a few remarks only to make regarding the inflammatory theory of decay, as I have previously clearly stated my views on this subject. Every practicing dentist must be impressed with the fact that there are very many peculiarities connected with caries of the teeth which directly contradict the idea of inflammatory action, and there are none as yet definitely established which agree with it. At the last Saratoga meeting, it was said that Dr. Miller had forgotten that there is a large amount of organic matter in the teeth, a very erroneous statement, as I have never lost sight of the fact for an instant, nor of the fact that there is also much organic matter in nails, hoofs, hair, wool, feathers, etc. We need other evidence than this, before we can call caries an inflammatory process.

Again, the mere detection of swelling or tumefaction of the dentinal fibrils is no evidence that caries is the result of inflammatory action. The custom of certain dental pathologists to assume that every variation from the normal detected in the tissue is of an inflammatory nature, is about as rational as it would be for the general histologist to ascribe the many post-mortem changes which take place in delicate tissues, while being prepared for examination, to inflammation. Experimenters have long ago shown that such post-mortem changes may take place in dentine, and I have produced them artificially. When Dr. Bodecker was in Europe, he examined a number of my preparations, and, in one instance in particular, he called attention to the absorption territories and swollen fibrils, not knowing that he had a case of artificial caries before him. He was much surprised on being told so, and said that I had "saved him an immense amount of labor."

I do not say that changes of an inflammatory character may not take place in the dentinal fibrils, as a result of the action of the caries-producing agents, or in connection with inflammation of the pulp. Indeed, it would be strange if the acids penetrating the dentine and bathing the fibrils did not produce some change in them. But it remains to be shown that in either case these changes either produce caries, or accelerate the process when once established.

I have no prejudice whatever against the inflammatory theory of decay. On the contrary, I would gladly welcome it, if it could be shown that there is at least a trace of truth in it. But at present there are too many and too great obstacles in the way. To say nothing of the facts that in caries the cardinal symptoms of inflammation are all wanting, that it is impossible to produce caries by those means which invariably produce inflammation of other tissues, that caries attacks pulpless and dead teeth as readily as living ones, and that caries can be produced artificially—aside from these facts there is not a little evidence which points to anything but inflammation as the cause of caries.

Anyone who has given time to the study of inflammation, particularly of bone or cartilage, will at once be impressed with the fact that there is not the slightest trace of similarity between it and *caries dentium.* Furthermore, the action of different coloring matters upon carious dentine furnishes information of considerable interest. For example : Picro-carmine colors the simply decalcified, otherwise unchanged basis-substance pink or red, while the distended tubules and the round or oval caverns filled with the debris and fungi (called by Abbott medullary elements), are stained yellow. This reaction is in marked contrast to that with pulp tissue, periodontium, bone, cartilage, etc., where changes of a truly inflammatory character have taken place.

Again, the so-called medullary elements are found in dead as well as in living teeth, and mostly in the outer layers of carious dentine which have lost all vitality, and cannot therefore be subject to inflammation. The deciding test for the correctness of any theory of caries is the possibility of its application in the direct production of caries, and if any advocate of the inflammatory theory can produce a speck of carious dentine as big as the point of a pin, within the next ten years, the inflammatory theory will be entitled to more consideration than it now merits. As stated above, I am ready at any time to acknowledge inflammatory action as an agent in the production of caries as soon as a single definite proof has been established in its favor. In a following number of this journal I shall consider the "toxic agents" and "digestive fluids" formed by certain of the micro-organisms of the human mouth.

## Figures and Tables

**Fig. 1. f1:**